# Quality of Type 2 Diabetes Management in the States of The Co-Operation Council for the Arab States of the Gulf: A Systematic Review

**DOI:** 10.1371/journal.pone.0022186

**Published:** 2011-08-04

**Authors:** Layla Alhyas, Ailsa McKay, Anjali Balasanthiran, Azeem Majeed

**Affiliations:** 1 Department of Primary Care and Public Health, Imperial College London, London, United Kingdom; 2 Department of Medicine, Imperial College London, London, United Kingdom; Universita Magna-Graecia di Catanzaro, Italy

## Abstract

Type 2 diabetes mellitus is a growing, worldwide public health concern. Recent growth has been particularly dramatic in the states of The Co-operation Council for the Arab States of the Gulf (GCC), and these and other developing economies are at particular risk. We aimed to systematically review the quality of control of type 2 diabetes in the GCC, and the nature and efficacy of interventions. We identified 27 published studies for review. Studies were identified by systematic database searches. Medline and Embase were searched separately (via Dialog and Ovid, respectively; 1950 to July 2010 (Medline), and 1947 to July 2010 (Embase)) on 15/07/2009. The search was updated on 08/07/2010. Terms such as diabetes mellitus, non-insulin-dependent, hyperglycemia, hypertension, hyperlipidemia and Gulf States were used. Our search also included scanning reference lists, contacting experts and hand-searching key journals. Studies were judged against pre-determined inclusion/exclusion criteria, and where suitable for inclusion, data extraction/quality assessment was achieved using a specifically-designed tool. All studies wherein glycaemic-, blood pressure- and/or lipid- control were investigated (clinical and/or process outcomes) were eligible for inclusion. No limitations on publication type, publication status, study design or language of publication were imposed. We found the extent of control to be sub-optimal and relatively poor. Assessment of the efficacy of interventions was difficult due to lack of data, but suggestive that more widespread and controlled trial of secondary prevention strategies may have beneficial outcomes. We found no record of audited implementation of primary preventative strategies and anticipate that controlled trial of such strategies would also be useful.

## Introduction

### The Type 2 Diabetes Mellitus problem

Diabetes mellitus is a chronic disease characterised by insufficient insulin production and/or insulin resistance. Through its various complications and a widespread high prevalence [1], diabetes mellitus is a major contributor to morbidity and mortality worldwide. Insulin resistance with a relative or real insulin deficiency is the hallmark of type 2 diabetes mellitus. Over the last 3–4 decades, the prevalence of type 2 diabetes has risen dramatically across the world [2], [3]. It currently accounts for over 90% of all diabetes cases [4]. Various factors including population growth, ageing, continued urbanisation and lifestyle modifications encouraging sedentary lifestyles and obesity, will lead to further increases in prevalence. Diabetes is a major public health issue, carrying huge societal and economic, as well as personal, costs and risks. This has been acknowledged by the United Nations through Resolution 61/225 (2006), which issued a call for Member States to implement strategies to address the burden of diabetes in their societies.

### Type 2 diabetes in the Gulf region

The states of The Co-operation Council for the Arab States of the Gulf (GCC) exhibit some of the highest rates of type 2 diabetes in the world. Five of the International Diabetes Federation's ‘top 10’ countries for diabetes prevalence in 2010 and in 2030 are projected to be in this region [1]. The anticipated prevalences for diabetes 2010–2030 in the Gulf countries are: United Arab Emirates (UAE) 18.7–21.4%, Kingdom of Saudi Arabia (KSA) 16.8–18.9%, Bahrain 15.4–17.3%, Kuwait 14.6–16.9% and Oman 13.4–14.9% [1]. The recent and rapid socio-economic development of the GCC countries has been associated with this rising prevalence. The International Diabetes Federation suggests that even in the absence of further economic development (that is, based on changes in population demography alone), the number of people with diabetes in its Middle East-North Africa region will increase 94% from 2010 to 2030. Only the Sub-Saharan African region is expected to see a greater increase in the number of cases of diabetes (98%) during this period [1].

### Responding to the type 2 diabetes problem

Many countries have responded to the concerns about type 2 diabetes by producing and implementing national diabetes programmes (at the suggestion of the World Health Assembly, aided and monitored by the International Diabetes Federation). The International Diabetes Federation suggests Oman, Kuwait and Bahrain have all implemented national diabetes programmes (with no data available for the UAE and KSA, and no national diabetes programme in Qatar) [1]. The UAE, however, published national guidelines in 2009 [5]. We have not been able to determine that the KSA has a national programme, but note that it produces by far the greatest research output on diabetes. For all countries, the extent and timings of programme implementation are unclear, and in many cases the content of the programmes also. Although the IDF suggests various dimensions that a national diabetes programme would ideally include, there are no particular suggested standards in any of these themes [1]. Although this reflects the need for locally tailored programmes, it perhaps also reflects that there are no standardised desired clinical outcomes, even in relatively well-studied populations. Both the extent and efficacy of current diabetes management in the GCC region is thus unknown.

### Review aims

The aim of this review was to examine the current quality of management of type 2 diabetes in the member states of the GCC. Unchecked, the chronic hyperglycaemia of diabetes is associated with various adverse macro- and micro- vascular outcomes. Glycaemic-, blood pressure- and lipid- control were used as indicator outcomes as they are relatively well established correlates of adverse vascular sequelae; preliminary searches suggested these were relatively frequently considered outcomes; and they are widely incorporated into national guidelines e.g. [6], [7], [8]. We aimed to, wherever possible, specify results according to age and gender, as evidence indicates that age/gender specified sub-populations with specific disease prevalences and characteristics or severity may exist, and thus that these populations may benefit from differential management strategies. Due to the heterogeneity of studies identified on preliminary searching, there was no anticipated meta-analysis.

## Methods

Ethical approval was not needed as this study was a systematic review, with no primary data collection.

### Review questions

A systematic literature search was carried out to identify information relevant to the following review questions:

1. How good is current control of type 2 diabetes in the GCC region, based on glycaemic-, blood pressure- and lipid- control indicators?
2. Have implemented strategies (including public health/preventative strategies) improved management of type 2 diabetes in GCC countries?

### Search

We developed a systematic review protocol (available from the authors on request) using the Centre for Reviews and Dissemination guidelines [9]. The Medline and Embase databases (via Dialog and Ovid, respectively; 1950 to July 2010 (Medline), and 1947 to July 2010 (Embase)) were searched separately on 15/07/2009 and the search was updated on 08/07/2010. The search was carried out using terms identified from PICOS deconstruction of the above review questions, and database- and manually- derived alternatives (see Appendix S1). Keywords used in the search strategies reflected the quality of management of type 2 diabetes and blood pressure, lipids and glucose in the GCC such as diabetes mellitus, non-insulin-dependent, hyperglycemia, hypertension, hyperlipidemia and Gulf States. The search strategy (available from the authors on request) was trialled, reviewed by independent professional colleagues (E.H, K.P), and updated (on 02/02/2010) before use. Further relevant studies were identified by searching the reference lists of the database-derived papers, contacting expert investigators, screening conference proceedings including those of The International Conference on Recent Advances in Diabetes Mellitus and Its Complications 2006 and Gulf Research Meeting 2010, citation searching and hand searching the available online contents of the International Journal of Diabetes and Metabolism and the Saudi Medical Journal, between the periods 1993–2009 and 2000–2010, respectively.

### Selection

**Figure 1:**
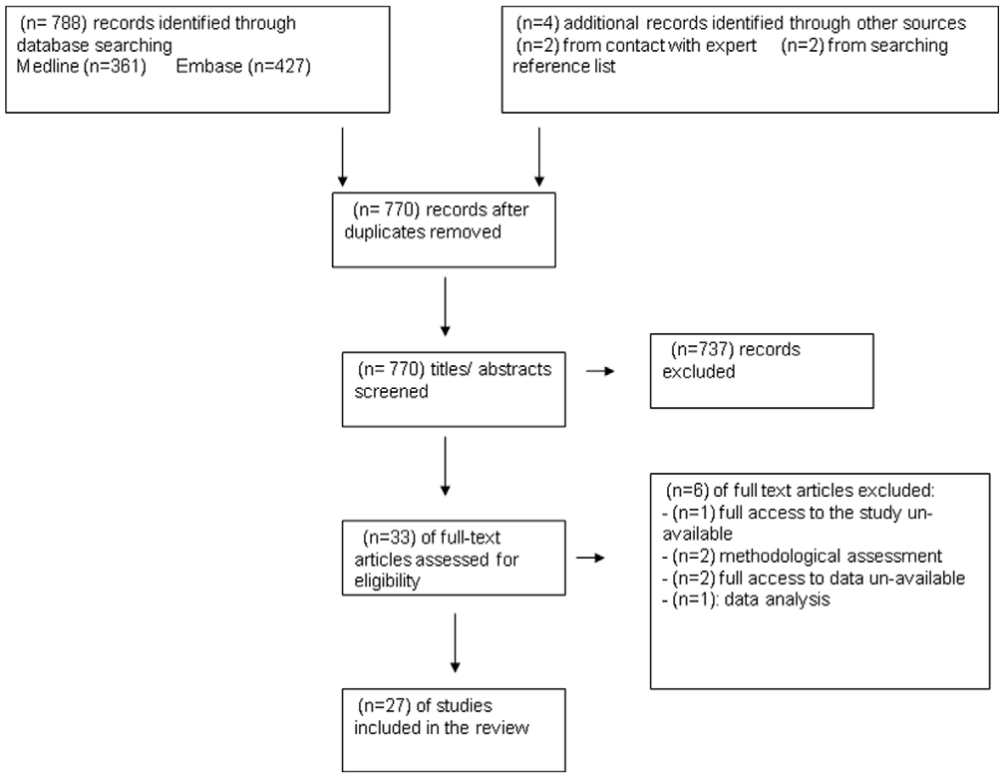
Flow chart study selection process.

The search yielded 788 studies. The titles and abstracts were evaluated by one reviewer to determine eligibility for full screening. Studies that utilised designs from a pre-determined list of acceptable methods - including randomized controlled trial and observational study (cross sectional, quasi-experimental and interventional) - were included. All studies wherein glycaemic-, blood pressure- and/or lipid- control were investigated (clinical and/or process outcomes) were eligible for inclusion. In addition, any study describing primary preventative measures was eligible. No limitations on publication type, publication status, study design or language of publication were imposed. However, we did not include secondary reports such as review articles without novel synthesis. The inclusion criteria demanded that the study population be people with diabetes (at least predominantly type 2; unless a study relating to primary prevention), and of a GCC country. All ages, sexes and ethnicities were included, resident and expatriate populations, urban and rural, of all socioeconomic and educational backgrounds. General population studies and studies at all healthcare levels were included. 33 studies were identified as suitable for full review, and were each considered by 2 reviewers. 6 studies were excluded, by consensus, either because data were not (fully) available, or because the reporting left us unable to assess, sufficiently, study quality (see Figure 1 and Figure S2).

### Data extraction/quality assessment

The data captured for each study included data relating to, (1) methods (study design, recruitment, measurement tools, analysis), (2) participant characteristics, (3) setting, and (4) outcomes (those observed, their definitions, results of analysis, length of follow-up). Study quality was assessed using a checklist adapted from the Centre for Reviews and Dissemination guidelines (see Appendix S2) [9]. Data extraction was performed, in duplicate, by two reviewers. Any disagreements were resolved by discussion with a third reviewer. Refer to Fig S1.

### Data synthesis

Data synthesis included summarising the results of the data extraction process, considering the strength of evidence relating to each of our questions, and examination of results inconsistent with our formed proposals. Synthesis was limited by the numbers of studies, particularly in consideration of the identified quasi-experimental studies (see ‘Results’), and thus for this set of data, description and discussion suffices.

## Results

We identified 27 journal-published studies for review: 21 cross-sectional and 6 quasi-experimental studies.

### 1. Cross-sectional studies

The cross-sectional studies included one undertaken in 1988/1989, 2 in the 1990s, the remainder from 2000 onwards. The studies were carried out in KSA [14], UAE [3], Bahrain [3] and Oman [1]. In all but one study, wherein subjects with diabetes were identified through a general population screening [20], studies were carried out in primary care or hospital environments. All involved retrospective review of patient records, and a very small minority included a prospective component. Identification of individuals with diabetes was in all cases by previous diagnosis. In some cases, diagnosis of type 2 diabetes was specified, otherwise the populations were mixed diabetic populations of predominantly type 2 diabetes. Sample size ranged from 30–1236.

We identified 15 studies of each of glycaemic- and blood pressure- control, and 11 of lipid control. In all cases, the lack of standardised targets for these outcome measures was reflected in a heterogeneous collection of definitions of control. Data that would allow comparison of subgroup outcomes were generally not available.

#### Glycaemic control

The identified studies of glycaemic control [10]–[24] are summarised in Table S1. One study investigated process measures alone (although several additional studies included these). 12/15 studies that reported clinical outcomes considered glycosylated haemoglobin A1c (HbA1c) levels, 6/15 fasting blood glucose (one fasting blood glucose as a sole measure), and 3/15 ‘post-prandial’ blood glucose levels (one post-prandial glucose and fasting blood glucose alone).

With regard to clinical outcomes, target levels of HbA1c were almost always <7%, whereas the definition of ‘poor control’ was more variable, but generally more than at least 8%. ‘Good control’ by fasting blood glucose and post-prandial blood glucose were <7 mmol/l and <9 mmol/l, respectively. Fasting blood glucose >8 mmol/L and post-prandial glucose >10/11 mmol/L were considered ‘poor control’. Process measures variably required documentation of fasting blood glucose/HbA1c testing within the study period, or within the previous 6 or 12 months.

Consistently, <50% of patients achieved target glycaemic control (e.g. HbA1c: <7% were achieved in: 20.6% [16]; 45% [17]; 33.3% [20]; 31.1% [22]; 27% [23]; 24% [24]). A group of privately-treated patients in KSA, wherein 60.9% achieved HbA1c <7% (cf. 11.5% in government hospital-treated patients) was an exception [21]. Plotting the values across time, there was no obvious indication of recently improving/declining control. Process measures were less commonly investigated, and of variable outcome (0.4–98% achieved).

#### Blood pressure control

The identified studies of blood pressure control [10], [12], [15], [17]–[20], [22]–[29] are summarised in Table S2. One study considered only process measures. Three studies provided only rates of hypertension (of variable definition) as an outcome. The remainder provide (at least) rates of ‘well-controlled’ blood pressure, of more consistent definition. Rates of poor blood pressure control were reported as either:

1. A basic record of ‘current’ rates of hypertension, or
2. Documentation of all (cumulative) rates of treated and untreated hypertension

There may therefore be discrepancies where hypertension assessment is not standardised and where cases of well-controlled hypertension exist. This hinders comparisons already complicated by differential lengths of diabetes diagnoses. It seems clear, however, that blood pressure targets, however described by the study authors, are far from met. The <130/<80 mmHg or <130/<85 mmHg targets were met in between 6.8% and 32% of patients with a history of hypertension, and between 14.2 and 42.1% of the remaining samples, with one exception. Target blood pressure was met in 83% of the sample of Afandi *et al* [17].

Rates of hypertension – of both cumulative and non-cumulative measures, and of various criteria (see Table S2) – were frequently between 30–60%. Although only recorded in 3 studies, documentation of blood pressure checks suggested they were rigorously carried out, with almost 100% documentation of blood pressure measurement achieved.

#### Dyslipidaemia

The identified studies of lipid control [10], [12], [15], [17]–[20], [22]–[24], [30] are summarised in Table S3. Again process outcomes were of varied definition and infrequently studied, but where investigated, documentation of measurement within the previous year was achieved in 97% [17], 93% [20], 87% [19] and 14% [23]. The latter outlying result is from the most recent study, where large proportions of people with diabetes had not been screened for diabetes complications and/or cardiovascular risk factors in the previous 12 months. Unfortunately, the exact cause of the low documentation was not determined as the study did not test the compliance of people with type 2 diabetes to regular screening.

The definitions of dyslipidaemia used were variable and utilised various aspects of the lipid profile. Low density lipoprotein was the most commonly used clinical outcome, with a consistently applied target of <2.6 mmol/L. This was met in approximately 30–50% of patients, including in the cases of populations being entirely with or entirely without a history of dyslipidaemia [24]. High density lipoprotein, total cholesterol and triglyceride levels were also used as measures of lipid control. Thresholds for dyslipidaemia were not consistent, yet where each indicator was used in isolation, rates of dyslipidaemia were: 27.9% [10], 30% [10], 72% [30], 63% [30], 44.6% [15], 44.6% [15], 76.4% [22] and 59.9% [22].

### 2. Quasi-experimental studies

The 6 quasi experimental studies identified [31]–[36] are summarised in Table S4. The studies were carried out between 1998 and 2007. There were 2 Saudi studies, 3 from the UAE, 1 from Kuwait. The study interventions included implementation of newly-designed diabetes clinics/services, or use of a flow sheet to guide management. There was no public health or primary preventative aspect to any of the interventions.

All studies were based in primary care, and based on populations previously diagnosed with diabetes. The samples are likely to contain a predominance of type 2 diabetes patients, except that of Udezue *et al* [33], which is likely to include a large proportion of type 1 diabetes patients (based on age at diagnosis). Where reported, the mean durations of diabetes diagnoses were several years.

The outcomes monitored were generally concerned with adherence to implemented guidelines, but three studies also monitored some clinical outcomes, including glycaemic control, throughout their duration. Generally, interventions successfully increased compliance with clinical guidelines and improved clinical outcomes, where monitored, over the duration of the study. The studies were followed up for periods of 1 year [36], 18 months [31], [35], 2 years [32], and 4 years [33], [34] post-intervention. Unfortunately, there are major limitations with all these studies. Only one study [31] included a control population, and in this case the physicians involved in writing the guidelines for the developed intervention were largely from the intervention group.

## Discussion

We found management of type 2 diabetes in the GCC region – based on glycaemic-, blood pressure- and lipid- control indicators – to be suboptimal. Almost universally, fewer than 50% of patients meet targets for these clinical outcomes. There were no clear differences between primary and secondary or tertiary care (although possibly blood pressure was better controlled in hospital settings).

The reviewed intervention studies were largely uncontrolled, and thus difficult to interpret. All strategies reviewed here did appear to improve outcomes, but involved multiple interventions and are likely to have been carried out against a background of evolving healthcare. No intervention studied included a primary preventative dimension.

Although we rate the quality of type 2 diabetes management in the GCC region as ‘poor’, the outcomes are similar to those reported from elsewhere. Due to the disparity in genetic and environmental contexts, type of health system, differences in intervention methods and management guidelines and target thresholds, we do not intend to suggest that any particular intervention method is similarly efficacious across regions. Nevertheless, we noted that for both clinical- and process- outcomes, similar results are reported for other countries in the region such Lebanon [37] and Egypt [38], [39], [40]. In comparison with a selection of reports from various levels of healthcare in the UK [8], [41], [42], USA [43], [44] and Australia [45], clinical outcomes in the GCC countries were generally lower, but this was not always so. Lipid control [8], [43], [44] and blood pressure control [43], [44], [45] were most frequently potentially comparable between these non-GCC countries and the studies reviewed here, but Grant *et al* [44] also report a 34% attainment of HbA1c levels <7%, which would be consistent with a number of the results from the GCC region. Notably, of the non-GCC region studies mentioned, this study includes perhaps the highest proportions of patients under relatively high level care. Although it may therefore underestimate outcomes more generally achieved, it may in fact be a better comparator for the mixed populations included in our review. We note also that in many cases the outcomes of our reviewed studies would satisfy the upper thresholds of the UK Quality and Outcomes Framework targets [8].

With regard to process measures, these were generally well met in all settings, but probably more so in the non-GCC developed regions, particularly for glycaemic control. Finally, and importantly, we note that with regard to intermediate outcomes of diabetes control, there has been evident progress in at least the UK and USA [8], [42], [43], which we have not observed here to be the case in the GCC region.

### Limitations of study

A major limitation on the strength of our conclusions lies in the heterogeneity of the reviewed studies. They were of varied populations, reported on variable outcome measures, were from various levels of healthcare provision and different countries (although were predominantly from some and notably did not include all GCC countries). Our outcomes of review are therefore necessarily of only a broad nature, and as expected, they were not appropriate for use in synthesis of outcomes with estimates of confidence.

All of our reviewed studies were published in English. Overall, the clarity of reporting in the reviewed papers was considered relatively low; considered so as it often hampered assessment of study quality. In a few cases, we excluded studies due to an inability to sufficiently assess study quality (see Fig. 1). Otherwise, we did not exclude studies based on quality, but noted some major limitations, particularly in the intervention studies. With regard to the cross-sectional studies, the relatively low numbers of papers returned by each search led to difficultly identifying inconsistencies versus widening accepted value ranges and extent of possible effects, and in turn difficulty considering the strength of our final proposals. Nevertheless, we feel the data are sufficient that we might comment on their potential implications for type 2 diabetes care in the GCC region.

### Implications

We believe – based on the mentioned studies from non-GCC countries and the intervention studies reviewed here – that the standards of diabetes care in the GCC region can be improved. Both of these sets of studies suggest that improved adherence to process measures would improve clinical outcomes. In defining these desired process outcomes, and the mechanisms to comply, it may be useful to consider some of the interventions implemented in the reviewed intervention studies. These could potentially be as effective as those implemented elsewhere, and there is a degree of overlap. For example, the use of patient education programmes, diabetes specialist nurses and self-glucose monitoring appear to be potentially useful and are relatively well developed components of systems elsewhere. Continued auditing of these and other interventions will be important. Standardising both the process and clinical measures for clinical use and for auditing would be useful to facilitate comparisons, although this has yet to be achieved elsewhere, and fixing standards may be difficult. A review of potentially useful and realistic standards for this region has not been achieved and would be helpful.

We also consider that there is a large role for primary prevention programmes in any new management strategy. It is unclear whether or not any such intervention has been trialled in this region, and a concerted/wide-reaching programme is probably essential for feasibility and success of diabetes management. Finally, we have not considered strategies likely to produce changes in diabetes management without being aimed *specifically* towards this (e.g. those implemented as part of the World Health Organization ‘Innovative Care for Chronic Conditions Framework’ adaptations in health systems associated with the shift towards management of chronic rather than acute diseases [46]), but it is anticipated that such changes will also be an important part of managing the diabetes burden in the GCC region.

### Conclusions

To our knowledge, this study is the first to systematically review the quality of diabetes care in the GCC region. We found management of type 2 diabetes, as indicated by three major intermediate outcome measures (glycaemic control, blood pressure and lipid profile), to be sub-optimal in the GCC countries. In addition, we found that in many of the reviewed studies, there were quality issues that impacted on their usefulness. We thus feel attention to the management of diabetes in this region needs to be improved, and that enhanced management must include better quality of research and production of valuable data.

With regard to specific management strategies, we have here reviewed several studies of interventions, which suggest a number of secondary prevention strategies that may help in raising the quality of management in this region. However, other forms of intervention – particularly primary prevention strategies, which have not been clearly implemented or audited – are also likely to be useful. We anticipate that co-ordinated implementation of locally-successful/targeted strategies may be particularly effective. Continued, high quality review of all forms of interventions in the GCC states would also be desirable.

## Supporting Information

Figure S1 **PRISMA 2009 checklist.**
(DOC)

Figure S2 **PRISMA 2009 flow diagram.**
(DOC)

Table S1 **Summary of glycaemic control.**
(DOCX)

Table S2 **Summary of BP control.**
(DOCX)

Table S3 **Summary of lipid control.**
(DOCX)

Table S4 **Summary of intervention studies.**
(DOCX)

Appendix S1 **Research questions for quality of type 2 diabetes management in the GCC countries using PICO.**
(DOCX)

Appendix S2 **Study quality assessment.**
(DOCX)
