# Supplementary material for: Quality of Type 2 Diabetes Management in the States of The Co-Operation Council for the Arab States of the Gulf: A Systematic Review
Source: PLoS One. 2011 Aug 4;6(8):e22186. doi: 10.1371/journal.pone.0022186 (PMC3150334; doi:10.1371/journal.pone.0022186)
Supplement: Figure S2 — PRISMA 2009 flow diagram. (DOC) [file pone.0022186.s002.doc]

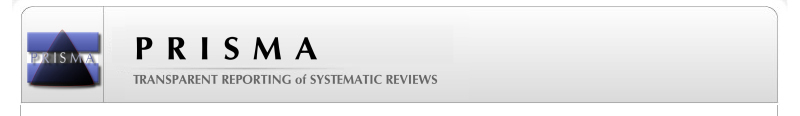
**Figure S2. PRISMA 2009 Flow Diagram.**

**Screening**

**Included**

**Eligibility**

**Identification**

Records identified through database searching
(n =788 )

Additional records identified through other sources
(n = 4 )

Records after duplicates removed
(n =770 )

Records screened
(n = 770 )

Records excluded
(n = 737 )

Full-text articles assessed for eligibility
(n =33 )

Full-text articles excluded, with reasons
(n = 6 )

Studies included in synthesis
(n =27 )
