# Supplementary material for: Quality of Type 2 Diabetes Management in the States of The Co-Operation Council for the Arab States of the Gulf: A Systematic Review
Source: PLoS One. 2011 Aug 4;6(8):e22186. doi: 10.1371/journal.pone.0022186 (PMC3150334; doi:10.1371/journal.pone.0022186)
Supplement: Table S1 — Summary of glycaemic control. (DOCX) [file pone.0022186.s003.docx]

**Table S1: Summary of glycaemic control**

| Ref/dates of study | Setting | Country | Sample size | Population characteristics | | | Outcomes (in bold) and results | | | | | | Study limitation |  |
| --- | --- | --- | --- | --- | --- | --- | --- | --- | --- | --- | --- | --- | --- | --- |
|  |  |  |  | % male | Age | | Glycaemic control indicators | | | Process outcomes (frequency of documentation) | | |  |  |
|  |  |  |  |  | Mean (SD) | Range | HbA1c levels (%) | FBG levels (mM) | Post-prandial glucose levels (mM) | HbA1c  levels | FBG  levels | Post-prandial glucose levels |  |  |
| (10)Famuyiwa et al / 1988 - 1989 | TC | KSA | 1000 | 54.2 |  | 1 - 98 | **> 8.8**: 77.2 % |  |  |  |  |  | -selection process and data collection not well described |  |
| (11)Al-Shammari et al / 1993 - 1994 | PC | KSA | 365 | NR | ‘All ages’ | | **< 8.1**: 60.6 %; **8.1 – 11**: 32.2 %; **> 11**: 7.1 % |  |  |  |  |  | -potential for selection of less severe cases  - potential for non-standardised treatment  -potential for non-standardised measurement of reported outcomes |  |
| (12)Khorsheed et al / 1998 - 2000 | PC | KSA | 138 | 69.6 | Mean:  Males: 49.7  Females: 53.4 |  |  |  |  | 73 % | 98 % |  | -very specific population **(**employees of National Guard)  -selection bias (single visits/those not seen after Jan 2008 excluded; potentially individuals with less severe disease included) |  |
| (13)Akbar/ 1999 - 2001 | UH | KSA | 443 | 49 | 54.8 ± 16.2 |  |  | **Mean** + SD: 9.7 ± 3.2 (38 % **< 7** : 11 % **7.1 – 8**: 51 %; **> 8**: 51 %) | **Mean** + SD: 13.75 ± 5.5 (22 % **< 9**: 7 % **9.1 – 10**; 71 % **> 10**) |  |  |  | -sample selection method not clear  -limitations of the study not discussed |  |
| (14)Al-Turki / 2000 - 2001 | PC | KSA | 1236 | 57.4 |  | < 15 to > 60 |  | **> 10**: 49.2 %; **7 - 10** : 28.9 %; **< 7**: 14 % |  | 0.4% | 92.1 % |  | -potential for lack of standardised measurements  -sampling methods not clear  -study limitations not discussed |  |
| (15)Al-Ghamdi / 2002 - 2003 | UH | KSA | 130 | 41.6 |  | 15 - 80 | **> 8**: 77 % | > 8 : 69 % | > 11: 69% | 49 % |  |  | -data analysis not well reported  -selection bias (some T1DM included)  -sample inclusion criteria unclear  - study limitations not discussed |  |
| (16)Al-Hussein / 2003-2004 | PC | KSA | 651 | 45.5 | 53.2 + 11.7 |  | **Mean** ± SD: 9.0 ± 2.0; **< 7**: 20.6 % | **Mean ± SD**: 9.9 ± 3.9 | **Mean ± SD**: 15.0 + 5.3 | 55.4 % | 64 % | 61 % | -potential for selection of less severe cases  -potential for non-standardised treatment, measurement of reported outcomes  - study limitations not discussed | |
| (17)Afandi et al / 2005 | TC | UAE | 30 | 40 | All > 18 | | **< 7**: 45 % |  |  | 97 % |  |  | -small sample size  -sampling method not clear  - potential for non-standardised measurement  - study limitations not discussed | |
| (18)Qari / 2005 | UH | KSA | 200*** | UH: 30  PH: 46 | UH: 47 + 14  PH: 49.4 + 13.7 |  | UH: **mean** ± SD: 7.8 ± 1.8; **< 6**: 24 %; **6 – 8**: 34 %  PH: **mean** ± SD: 7.8 ± 1.78; **< 6**: 14 %; **6 – 8**: 40 % |  |  |  |  |  | -selection method of hospitals unclear  -study limitations not discussed | |
| (19)Kharal et al / 2005- 2006 | TC | KSA | 1188 | 38.5 | All ≥ 30 years | | **Mean** ± SD: 9 ± 2; **<** 7: 21.8 % | **Mean**: 10 ± 4.2; **< 7**: 25.0 % |  | 81 % | 95 % |  | -specific population (Saudi national guards and their dependents) | |
| (20)Saadi et al/2005 - 2006 | GP | UAE | 245 | 44.9 |  | 18 to > 70 | **<7**: 33.3 % |  |  | (within 1 year): 91 % |  |  | -potential for under-/over-estimation of DM diagnosis (10.2%) based on disease reporting rate  - low % of subjects from sampled households (2455) underwent testing | |
| (21)Al-Shaikh / Not reported | UH | KSA | 392* | UH: 6.5  PH: 57.8 | (UH): 46.3  (PH): 46.1 |  |  | UH: **mean**: 9.89; **< 7**: 11.5 %  PH: **mean**: 7.1; **< 7**: 60.9 % | UH: **Mean**: 10.5  PH: **Mean**: 6.18 |  |  |  | -sample selection process not reported  -extent of co-morbidities e.g. smoking status not noted  -unclear statistical tests used | |
| (22)Al-Kaabi et al / 2006 | PC | UAE | 409** | 39 | 51.44 + 11.2 |  | **< 7**: 31.1 %; **7 – 8**: 19.6 %; **8 – 9**: 16.7 %; **> 9**: 32.6 % |  |  |  |  |  | -limited details re. sampling process | |
| (23)Al-Elq / 2006 | PC | KSA | 353 | NR | 51.6 + 10.8 |  | **Mean** + SD: 8.2 + 1.89; **< 7**: 27 % |  |  | (within 6 months): 81 % |  |  | **-** characteristics of population not well described  - non-standardised lab. assays  - lack of assessment of DM complications  -lack of evaluation of barriers that prevented achievement of various targets  - lack of calculation for suggested direct and indirect economic burdens of DM | |
| (24)Eledrisi et al / Not reported | Unclear | KSA | 1107 | 45.3 | All > 18 | | **<7**: 24 % ; **> 9.5**: 28.5 % |  |  |  |  |  | **-** potential lack of standardised measurement/reporting  -selection process unclear | |

Summary of cross-sectional studies investigating glycaemic control in diabetic patients in the GCC region.

PC = primary care; SC = secondary care; TC = tertiary care; UH = university hospital; PH = private hospital; GP = diabetic patients identified in cross-sectional study of general population; NR = not reported

* For government hospital sample n = 200; for private hospital sample n = 192; **204 SC patients, 205 PC patients; ***n = 100 for each hospital
