# Supplementary material for: Quality of Type 2 Diabetes Management in the States of The Co-Operation Council for the Arab States of the Gulf: A Systematic Review
Source: PLoS One. 2011 Aug 4;6(8):e22186. doi: 10.1371/journal.pone.0022186 (PMC3150334; doi:10.1371/journal.pone.0022186)
Supplement: Table S2 — Summary of BP control. (DOCX) [file pone.0022186.s004.docx]

**Table S2: Summary of BP control**

| Ref/dates of study | Setting | Country | Sample size | Population characteristics | | | | BP control indicators results (units of all BP measurements = mmHg) | | | Process outcomes  (Frequency of BP documentation) | Study limitations |
| --- | --- | --- | --- | --- | --- | --- | --- | --- | --- | --- | --- | --- |
|  |  |  |  | % male | Age | | Additional information | BP<130/80 | BP<130/85 | BP>130/80 |  |  |
|  |  |  |  |  | Mean (SD) | Range |  |  |  |  |  |  |
| (10)Famuyiwa et al / 1988 - 1989 | TC | KSA | 1000 | 54.2 |  | 1 - 98 | 77.7 % Saudi nationals |  |  |  |  | -sample selection method not clear  -data analysis not well described  (variable n-numbers for each outcome)  - unconventional definition of overweight/obesity  -limitations of the study not discussed |
| (12)Khorsheed et al / 1998 - 2000 | PC | KSA | 138 | 69.6 | Mean (males): 49.7  Mean (females): 53.4 |  | Saudi nationals |  |  |  | 100 % | - results may not be generalisable (population **=** employees of National Guard)  -potential for selection bias (single visits/those not seen after Jan 2008 excluded)  -potentially individuals with less severe disease selected  -limitations of the study not discussed |
| (25)Sequeira et al / 2001 | PC | Bahrain | 266 | 30.8 | 58.4 + 10.8^***^ |  | Hypertensive population |  | 9.8 %; |  |  | **-** potential for selection of less severe cases  -limited data on population characteristics (e.g. ethnicity and co-morbidities) |
| (26) Al-Khaja et al/ 2001 | PC | Bahrain | 357 | GP clinics: 27.2  Diabetic clinics: 34.5 | GP clinics: 58.1 + 10.5  Diabetic clinics: 54.8 + 10.8 |  | Hypertensive population |  | Diabetic clinic6.8 %  GP Clinic: :10 % |  |  | -limited data on population characteristics (e.g. co-morbidities, ethnicity, BMI)  -potential for selection bias (diabetic clinics for more severe cases; patients at GP clinics older)  -study limitations not discussed |
| (15)Al-Ghamdi / 2002 - 2003 | UH | KSA | 130 | 41.6 |  | 15 - 80 | 69% non-Saudi |  |  | **BP** > 140/90: 41.5 % |  | -data analysis not well reported  -selection bias (some T1DM included) |
| (27)Al-Khaja et al/ Not reported | PC | Bahrain | 220 | 36.4 | 54.9 + 10.7 |  | Hypertensive population |  | 7.5 % |  |  | **-** control of DM not included as hypertensive population only  -study limitations not discussed |
| (28)Al-Shehri/ 2003 – 2004 | PC | KSA | 403 | 55.8 |  | 29 - > 60 | 98.6 % Saudi; military personnel and dependants | 14.2 % |  |  |  | - results may not be generalisable (population = attendees of King Fahd Military Hospital)  -study limitations not discussed |
| (18)Qari / 2005 | UH | KSA | 200* | UH: 30  PH: 46 | UH: 47 + 14  PH: 49.4 + 13.7 |  | UH: 51 % Saudi  PH:62 % Saudi |  |  |  |  | -limited data re study population |
| (17)Afandi et al/ 2005 | TC | UAE | 30 | 40 | All > 18 | |  |  |  |  | at most recent appointment: 100 % | -small sample size  -sampling process not clear |
| (19)Kharal et al/ 2005- 2006 | TC | KSA | 1188 | 38.5 | All ≥ 30 | | Saudi National Guard employees and dependants | 39.1 % |  |  | 99 % **documented BP** result during period of interest | - results may not be generalisable (sample from King Fahd Military Hospital )  -lack of data re retinopathy screening, foot examination and neuropathy |
| (20)Saadi et al /2005 - 2006 | GP | UAE | 245 | 44.9 |  | 18 to > 70 | UAE nationals, urban residents | 42.1% |  |  |  | -sampling process not well described |
| (22) Al-Kaabi et al/ 2006 | PC | UAE | 409** | 39 | 51.44 + 11.2 |  | 50.4 % illiterate |  |  | 53.7 % |  | -sample selection method not clear  -study limitations not discussed |
| (23)Al-Elq  / 2006 | PC | KSA | 353 | NR | 51.6 + 10.8 |  | 84 % 'Arab/Oriental/ Persian'; 22 % literate; 63 % in full time employment | 16 % |  |  |  | -non-standardised lab. assays  - lack of assessment of DM complications  -lack of evaluation of barriers preventing achievement of various targets  - lack of calculation for suggested direct and indirect economic burdens of DM |
| (24)Eledrisi et al / Not reported | 'outpatient clinics' | KSA | 1107 | 45.3 | All > 18 | | 48.5 % history of HTN | 32% |  |  |  | **-** potential lack of standardised measurement/reporting  -sample selection method unclear |
| (29)El-shafie et al / 2006 - 2007 | UH | Oman | 210 | 28.6 | 53.7+ 9.1 |  | Hypertensive population | 34.4/76.1% |  | 41.6/7.7% |  | - sampling method not clear  - results may not be generalisable (sample from Sultan Qaboos University Hospital)  -limitations of the study not clear |

Summary of cross-sectional studies investigating BP control in diabetic patients in the GCC region.

PC = primary care; SC = secondary care; TC = tertiary care; UH = university hospital; PH = private hospital; GP = diabetic patients identified in cross-sectional study of general population; NR = not reported

* n = 100 for each hospital; **204 SC patients; 205 PC patients; ***data for final sample not reported;

^†^HTN = previous diagnosis/treatment or BP > 160/95 if patient > 40 years/ > 140/90 if patient < 40 years; ^††^definition of HTN not reported; ^†††^HTN = BP > 140/90 mmHg or antihypertensive medication; ^o^ HTN = systolic BP > 140 mmHg and/or diastolic BP > 90 mmHg or antihypertensive medication; ^oo^ HTN = BP > 140/90
