# Supplementary material for: Quality of Type 2 Diabetes Management in the States of The Co-Operation Council for the Arab States of the Gulf: A Systematic Review
Source: PLoS One. 2011 Aug 4;6(8):e22186. doi: 10.1371/journal.pone.0022186 (PMC3150334; doi:10.1371/journal.pone.0022186)
Supplement: Table S3 — Summary of lipid control. (DOCX) [file pone.0022186.s005.docx]

**Table S3: Summary of lipid control**

| Ref/dates of study | Setting | Country | Sample size | Population characteristics | Lipid control indicators  (Levels of TC, LDL, HDL, TG measurement) | | | | Process outcomes  (Frequency of lipid measurement documentation) | Study limitations |
| --- | --- | --- | --- | --- | --- | --- | --- | --- | --- | --- |
|  |  |  |  |  | TC | LDL | HDL | TG |  |  |
| (10)Famuyiwa et al / 1988 - 1989 | TC | KSA | 1000 | 54.2 % male; age range: 1 - 98 years; 77.7 % Saudi | 6.2 mM: 27.9 % |  |  | > 2.3 mM: 30.0 % |  | -selection process and data collection not well described  -unconventional definition overweight/obesity  -study limitations not discussed |
| (12)Khorsheed et al / 1998 - 2000 | PC | KSA | 138 | 69.6 % male; mean age males: 49.7 years; mean age females: 53.4 years; Saudi nationals |  |  |  |  | 69 % | - results may not be generalisable **(**sample = employees of National Guard)  -selection bias (single visits/those not seen after Jan 2008 excluded )  -potentially individuals with less severe disease selected |
| (30)Akbar et al/ 2000 - 2001 | UH | KSA | 202 | 50 % male; mean age ± SD: 59.9 ± 12.9 years; dyslipidaemic population |  | < 2.6 mM: 31 % | > 1.1 mM: 28 % | < 1.7 mM: 37 % |  | -interview questions unclear, and results not discussed  -study limitations not discussed |
| (15)Al-Ghamdi et al / 2002 - 2003 | UH | KSA | 130 | 41.6 % male; ages: 15 - 80 years; 69% non-Saudi | > 5.2 mM : 55.4% |  |  | > 2.3 mM: 55.4 % |  | -data analysis not well reported  -selection bias (some T1DM included) |
| (18)Qari / 2005 | UH/PH | KSA | 200* | UH:30 % male; mean age + SD: 47 + 14 years; 51 % Saudi  PH: 46 % male; mean age: 49.4 + 13.7 years; 62 % Saudi |  |  |  |  |  | -limited data re. study population |
| (17)Afandi et al / 2005 | TC | UAE | 30 | 40 % male; ages: > 18 years |  |  |  |  | 97% (within 1 year) | -small sample size  -sampling process not clear |
| (19)Kharal et al / 2005- 2006 | TC | KSA | 1188 | 38.5% male, age: ≥ 30 years; Saudi National Guard employees + dependants |  | < 2.6 mM: 55.5 % |  |  | **Documented LDL** measurement within period of interest: 87 % | -specific population (Saudi National Guard and dependents) |
| (20)Saadi et al/2005 – 2006 | GP | UAE | 245 | 44.9 % male; ages: 18 to > 70 years; UAE nationals, urban residents |  | < 2.6 mM: 30.8% |  |  | **Documented cholesterol** measurement within year: 93 % | -sampling process not well described |
| (22)Al-Kaabi et al / 2006 | PC | UAE | 409** | 39 % male; mean age + SD: 51.44 + 11.2 years; 50.4 % illiterate |  | > 2.5 mM: 78.6 % | < 1mM: 76.4 % | > 1.7 mM: 59.9 % |  | -limited details re. sampling process |
| (23)Al-Elq et al / 2006 | PC | KSA | 353 | Sex ratio not reported; mean age ± SD: 51.6 ± 10.8 years; 84 % 'Arab, Oriental, or Persian'; 22 % literate; 63 % in full time employment |  | > 2.6 mM: 65 % |  |  | within year: 14 % | **-** characteristics of population not well described  -non-standardised lab. assays  - lack of assessment of DM complications  - lack of evaluation of barriers preventing achievement of various targets  - lack of calculation for suggested direct and indirect economic burdens of DM |
| (24)Eledrisi/ Not reported | 'outpatient clinics' | KSA | 1107 | 45.3 % male; ages: > 18 years; Saudi nationals; 70 % history of (treated) dyslipidaemia |  | -< 2.6 mM: 50.5 % (patients with history of dyslipidaemia)  -> 3.38 mM: 17.6 % (patients without history of dyslipidaemia) |  |  |  | **-** potential lack of standardised measurement/reporting  -selection process unclear |

Summary of cross-sectional studies investigating BP control in diabetic patients in the GCC region.

PC = primary care; SC = secondary care; TC = tertiary care; UH = university hospital; PH = private hospital; GP = diabetic patients identified in cross-sectional study of general population

* n = 100 for each hospital; **204 SC patients; 205 PC patients
