# Supplementary material for: Quality of Type 2 Diabetes Management in the States of The Co-Operation Council for the Arab States of the Gulf: A Systematic Review
Source: PLoS One. 2011 Aug 4;6(8):e22186. doi: 10.1371/journal.pone.0022186 (PMC3150334; doi:10.1371/journal.pone.0022186)
Supplement: Table S4 — Summary of intervention studies. (DOCX) [file pone.0022186.s006.docx]

**Table S4: Summary of intervention studies**

| Ref/dates of study | Country | Sample size | Population characteristics | Intervention | Outcomes observed | Main outcomes | Study limitations |
| --- | --- | --- | --- | --- | --- | --- | --- |
| (31)Reed et al 2001 | UAE | 219 | Control group: 52.3 % male; mean age + SD: 53.6 + 10.9 years; 84.6 % UAE nationals; mean + SD years education: 3.09 + 4.49 years  Intervention group: 64.5 % male; mean age + SD: 49.4 + 11.7 years; 83.0 % UAE nationals; mean + SD years education: 3.12 + 4.87 years | Guideline implementation, chronic care clinics established, patient and provider education, improved clinical recording | Adherence to guidelines  Clinical outcomes: HbA1c, BP, lipid levels  Patient knowledge re. DM and patient satisfaction | Some baseline differences in clinical outcomes between groups; aadherence to guidelines improved with intervention; higher satisfaction levels in intervention group | -Potential for different clinics to attract different population types (e.g. in terms of socioeconomic status, ethnicity)  -Potential for different definitions, instruments and processes between clinics |
| (32)Andrews/ 1998 - 2000 | UAE | 721* | 43.6 % of patients who attended > once male; mean + SD age of patients who attended > once: males 56.8 + 13.2 years, females 53.7 + 12.5 years | ‘Mini clinic’ (provider education, computer-assisted record keeping, guideline implementation) | Adherence to guidelines; HbA1c levels | Significant ↓ in HbA1c over 12 – 18 months, same as entry at 2 years; ⭡compliance with guidelines | -no controls  -analysis not fully discussed |
| (33)Udezue et al/ 1998-2002 | KSA | 105 | 48.6 % male; ages: 14 - 20 years; employees of Saudi Aramco Medical Services Organization and dependants | ‘Young diabetes clinic’ (lifestyle, medication and other education) | Assessment of management via monitoring:  Compliance with attendance, use of glucose meters; HbA1c levels; eye, vascular and neurological examination results | ⭡ appointment attendance and use of glucose meters; no significant improvement in HbA1c levels; no patients developed retinopathy or neuropathy | - no control group  - analysis not well described; co-morbidities and types of DM not stated  - specific population: young people |
| (34)Al-Adsani et al/ 2001-2003 | Kuwait | 250 | Demographics of sample not reported | Clinical guidelines developed; training courses; implementation of auditing | Adherence to guidelines | ⭡ use of appointment/filing systems; various clinical measures, examinations and smoking assessments achieved more frequently | -no controls  - characteristics of population studied not clear  - selection of records not clear |
| (35)Khattab et al/ 2002 - 2005 | UAE | 2548** | 51.8 % male; mean age + SD: 55.3 + 11.6 years; 66 % UAE nationals; 90 % T2DM diagnosis | Clinical guidelines and information systems developed, diabetes nurse practitioners introduced; DM ‘teams’ formed; implementation of auditing | Clinical outcomes: HbA1c, BP, lipid levels  Documentation of BMI, smoking status, fundoscopy referral | Significant ↓ in HbA1c, systolic BP and LDL over study period; ⭡ documentation of HbA1c, BP, LDL, BMI measurements, smoking status and fundoscopy referral between 1^st^ and 2^nd^ audits |  |
| (36)Moharram et al/ 2006-2007 | KSA | 371 | 46 % male; mean age + SD: 55 + 5.8 years; military personnel and dependants | Flow sheet to guide management | Adherence to guidelines | ⭡ documentation of various clinical measures and examinations; ⭡ patient education and dietician referral | - no controls  - very specific population (military personnel) |

Summary of outcomes of trialled interventions aiming to improve control of DM in UAE, KSA and Kuwait. All studies were carried out in primary care settings.

* n fell over follow up period to n = 45 in males at 21 - 27 months; **n at study outset/for 1^st^ audit; n for 2^nd^ audit = 1234
