# Supplementary material for: Quality of Type 2 Diabetes Management in the States of The Co-Operation Council for the Arab States of the Gulf: A Systematic Review
Source: PLoS One. 2011 Aug 4;6(8):e22186. doi: 10.1371/journal.pone.0022186 (PMC3150334; doi:10.1371/journal.pone.0022186)
Supplement: Appendix S1 — Research questions for quality of type 2 diabetes management in the GCC countries using PICO. (DOCX) [file pone.0022186.s007.docx]

**Appendix S1: Research questions for quality of type 2 diabetes management in the GCC countries using PICO.**

1. **Research question:** How good is current control of people with type 2 diabetes in the GCC regions, based on glycaemic, blood pressure- and lipid- control indicators?
   - 1. **The question as a testable hypothesis:** in people with type 2 diabetes in the GCC **(P)**, do the indicated levels of blood glucose, pressure and lipids control **(I)** results in good diabetes management **(O)?**

AND

AND

| **Patient/Population** | **Intervention** | **Outcomes** |
| --- | --- | --- |
| **People with type 2 diabetes mellitus** in the **GCC** | Level of **blood glucose, pressure and lipids** Control | Diabetes **management** |
| **Alternative Words** | | |
| **Type 2 diabetes mellitus:** Diabetes mellitus, Non-insulin dependent diabetes mellitus, T 2 DM, Diabetes type 2, Type 2 diabetes, diabetics  **GCC:** Qatar, United Arab Emirates UAE, Kingdom of Saudi Arabia KSA, Kingdom of Bahrain, Sultanate of Oman, Kuwait | **Blood glucose:** high blood glucose, hyperglycaemia, hyperglycemia, HbA1c, glyclated haemoglobin levels , Fasting blood glucose levels, FBG, Postprandial blood glucose level  **Blood pressure:** High blood pressure, diastolic/ Systolic blood pressure, hypertension, BP, HTN ,hypertensan  **Blood lipid:** High blood lipid, hyprcholesterolaemia, hypercholesterol, hyperlipidemia, hyperlipidemaemia, hyperlipidemic, hypertriglycerdemia | **Management:** control, reduction, improvement, enhancement |

1. **Research question:** Have implemented strategies (including public health/ preventive strategies) improved management of type 2 diabetes in the GCC countries?
   - 1. **The question as a testable hypothesis:** in people with type 2 diabetes in the GCC **(P),** do the implemented strategies (public health/ preventive) **(I),** Improve management of type 2 diabetes **(O)?**

| **Patient/Population**  AND | **Intervention**  AND | **Outcomes** |
| --- | --- | --- |
| **People with type 2 diabetes mellitus** in the **GCC** | Implemented strategies (public health/ preventive) | **Management** of type 2 diabetes |
| **Alternative Words** | | |
| **Type 2 diabetes mellitus:** Diabetes mellitus, Non-insulin dependent diabetes mellitus, T 2 DM, DM/,Diabetes type 2, Type 2 diabetes, diabetics  **GCC:** Qatar, United Arab Emirates UAE, Kingdom of Saudi Arabia KSA, Kingdom of Bahrain, Sultanate of Oman, Kuwait | National diabetes programmes, national guidelines, prevention programmes, public health programmes, diabetes management programmes | **Management:** control, reduction, enhancement, improvement |
