# Supplementary material for: Quality of Type 2 Diabetes Management in the States of The Co-Operation Council for the Arab States of the Gulf: A Systematic Review
Source: PLoS One. 2011 Aug 4;6(8):e22186. doi: 10.1371/journal.pone.0022186 (PMC3150334; doi:10.1371/journal.pone.0022186)
Supplement: Appendix S2 — Study quality assessment. (DOCX) [file pone.0022186.s008.docx]

| **Ref/dates of study** | **Quality assessment checklist (1)** |
| --- | --- |
| (10)Famuyiwa et al / 1988 - | 1-Y, 2-Y, 3-Y, 4-N, 5-Unclear, 6-N, 7-NA |
| (11)Al-Shammari et al / 1993 - 1994 | 1-N, 2-N, 3- Unclear, 4- N, 5-N, 6-Y, 7-NA |
| (12)Khorsheed et al / 1998 - 2000 | 1-N, 2-Incomplete, 3- Y, 4- Not well described, 5- Unclear, 6-N, 7-NA |
| (13)Akbar/ 1999 - 2001 | 1-Y, 2-Y, 3-Y, 4-Y, 5-N, 6-N, 7-NA |
| (14)Al-Turki / 2000 - 2001 | 1-Y, 2-Unclear, 3- Y, 4-N, 5-Unclear, 6-N, 7- NA |
| (15)Al-Ghamdi / 2002 - 2003 | 1-N, 2- Y, 3-Y, N, 4- Unclear, 5- N, 6- N, 7-NA |
| (16)Al-Hussein / 2003-2004 | 1-Y, 2-incomplete, 3- Y, 4- Y, 5- Unclear, 6- N, 7-NA |
| (17)Afandi et al / 2005 | 1-Y, 2-Y, 3-Y, 4- Unclear, 5- Unclear, 6- N, 7- NA |
| (18)Qari / 2005 | 1-Y, 2-Y, 3- Y, 4- Y, 5- Partially, 6-N, 7-NA |
| (19)Kharal et al / 2005- 2006 | 1-Y, 2-Y, 3-Y, 4-Y, 5-Y, 6-partially, 7-NA |
| (20)Saadi et al/2005 - 2006 | 1-Y, 2-Y, 3-Y, 4-Y, 5-N, 6- Y, 7-NA |
| (21)Al-Shaikh / Not reported | 1-Y, 2-Partially , 3- Unclear, 4-Unclear, 5-N, 6- N, 7-NA |
| (22)Al-Kaabi et al / 2006 | 1-Y, 2-Y, 3- Y, 4- Y, 5-Unclear, 6 –Partially, 7-NA |
| (23)Al-Elq / 2006 | 1-Y, 2-Y, 3-Y, 4-Y, 5- Unclear, 6-Y, 7-NA |
| (24)Eledrisi et al / Not reported | 1-Y, 2-Y, 3-Y, 4- Y, 5-Unclear, 6-N, 7- NA |
| (25)Sequeira et al / 2001 | 1-N, 2-Y, 3-Y, 4-Y, 5-N, 6-Partially, 7- NA |
| (26) Al-Khaja et al/ 2001 | 1-Y, 2-Y, 3-Y, 4-Y, 5-N, 6-N, 7-NA |
| (27)Al-Khaja et al/ Not reported | 1-Y, 2-Y, 3-Y, 4-Y, 5-N, 6-N, 7-NA |
| (28)Al-Shehri/ 2003 – 2004 | 1-Y, 2-Y, 3-Y, 4-Y, 5-N, 6-N, 7-NA |
| (29)El-shafie et al / 2006 - 2007 | 1-Y, 2- Y, 3-Y, 4-Y, 5- N, 6- N, 7-NA |
| (30)Akbar et al/ 2000 - 2001 | 1-Y, 2-Y, 3-Y, 4-Y, 5- N, 6-N, 7-NA |
| (31)Reed et al 2001 | 1-Y, 2-Y, 3- Y, 4- Y, 5- Unclear, 6-Y, 7- NA |
| (32)Andrews/ 1998 - 2000 | 1-Y, 2-N, 3- Unclear, 4-Y, 5-Unclear, 6-N, 7-NA |
| (33)Udezue et al/ 1998-2002 | 1-Y, 2- Partially stated, not entirely appropriate , 3- not entirely appropriate, 4- Unclear, 5-N, 6- N, 7-NA |
| (34)Al-Adsani et al/ 2001-2003 | 1-Y, 2, Unclear, 3-Unclear, 4-Y, 5-Unclear, 6-Y, 7-NA |
| (35)Khattab et al/ 2002 - 2005 | 1-Y, 2-Y, 3-Y, 4-Y, 5-N, 6-N, 7-NA |
| (36)Moharram et al/ 2006-2007 | 1-Y, 2-Y, 3-Y, 4-Y, 5-Y, 6-Y, 7-NA |

**Appendix S2: Study quality assessment.**

Quality assessment checklist (1)

1. ^Was the aim of the study stated clearly?^
2. ^Was the methodology stated? And was it appropriate?^
3. ^Were appropriate methods used for data collection and analysis?^
4. ^Was the data analysis sufficiently rigours?^
5. ^Were preventive steps taken to minimize bias?^
6. ^Were limitations of the study discussed?^
7. ^In systematic review, was search strategy adequate and appropriate?^
